# Couvade Syndrome: Origin, Characterization, and Frequency

**DOI:** 10.1192/j.eurpsy.2022.1398

**Published:** 2022-09-01

**Authors:** J. Sá Couto, M. Pão Trigo, B. Da Luz, J. Rodrigues, T. Ventura Gil

**Affiliations:** Centro Hospitalar Universitário do Algarve, Departamento De Psiquiatria E Saúde Mental, Unidade De Faro, Faro, Portugal

**Keywords:** Couvade, Somatic, Cultural, Pregnancy

## Abstract

**Introduction:**

The word *couvade* originated from the French verb *couver*, meaning to hatch, nest, or brood. Custom of Couvade or Couvade Syndrome (CS) is a poorly understood phenomenon observed since ancient times, in which the expectant father experiences somatic and psychological symptoms of pregnancy.

**Objectives:**

Defining what is CS. Identifying possible origin. Hypothesizing causes. Identifying CS frequency.

**Methods:**

*PubMed* database search, with *“Couvade syndrome”* keyword expression. Seven articles were selected among the best matches. Reference lists of articles were reviewed to identify additional articles.

**Results:**

Currently, there are several views on this phenomenon, including religious, cultural, medical, psychoanalytic, and psychological. CS is used in Psychiatry to describe somatic symptoms resembling pregnancy and/or childbirth in expecting fathers, such as weight gain, diarrhea or constipation, toothache, and headache. Lipkin and Lamb (1982) studied 300 couples from New York: they diagnosed Couvade Syndrome in 22,5% of fathers. Nevertheless, Brennan et al. (2007) found different incidence rates of CS diagnose in different areas of the world: 20% in Sweden; 25–97% in United States; 61% in Thailand; 68% in China; 35% in Russia.

**Conclusions:**

Whether CS constitutes a disease entity, or it should be considered a ritual or custom remains a matter of debate. Different rates of CS around the globe may indicate that culture plays an important role. It may be a way for fathers-to-be to cope with changes imposed by pregnancy in the mother and in the couple. Overall, it is a fascinating intersection between the physiological and psychological realms.

**Disclosure:**

No significant relationships.

